# A Collaborative Cocurricular Undergraduate Research Experience on Sustainable Materials: Analysis of Biochar Using the Boehm Titration and Spectroscopic Techniques

**DOI:** 10.1021/acs.jchemed.4c01110

**Published:** 2025-02-07

**Authors:** Rachel Breen, Conor Goggin, Justin D. Holmes, Gillian Collins

**Affiliations:** † School of Chemistry, University College Cork, Cork T12 YN60, Ireland; ‡ AMBER Centre, Environmental Research Institute, University College Cork, Cork T23 XE10, Ireland

**Keywords:** biochar, Boehm titration, spectroscopy, sustainable materials, undergraduate research experience, cocurricular activity, research skills, inquiry-based learning, surface chemistry

## Abstract

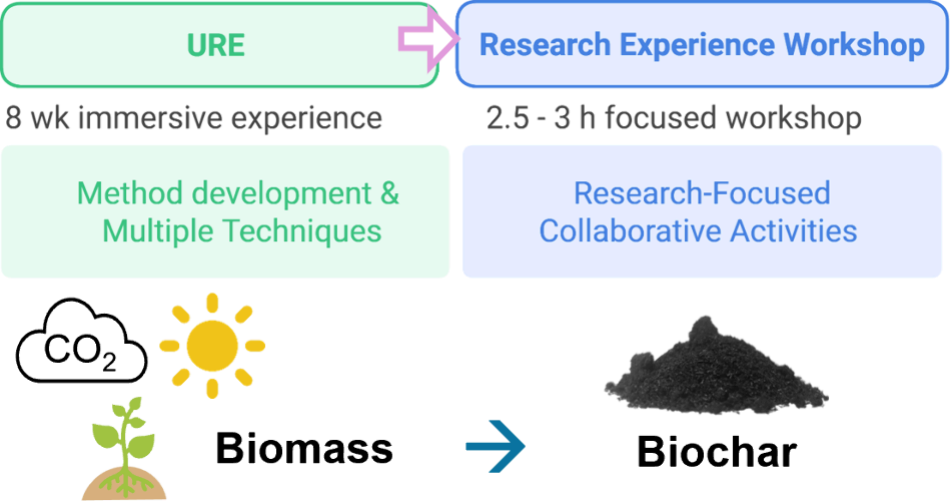

The use of renewable feedstocks such as biomass aligns with global priorities such as sustainability and climate change. Integrating these materials into experiments helps students understand the real-world relevance of chemistry to addressing environmental challenges. Here, we show that the use of biochar as a renewable feedstock provides an inquiry-based laboratory activity that gives students the opportunity to engage in an authentic investigative process. This activity describes a cocurricular summer workshop carried out with undergraduate students who had no prior research experience. The activity combined the Boehm titration as a chemical method for the analysis of biochar coupled with spectroscopic techniques. The workshop was designed to be collaborative in nature, where students collectively contributed to the overall experimental results and discussion. The motivation for the activity stems from a student undertaking a longer Undergraduate Research Experience (URE) in the form of a summer research placement and based on this work designing a research experience workshop that could be rolled out to benefit a larger number of students. We believe this approach of using longer or individual UREs to develop research-focused initiatives could be readily adopted by other UREs to promote and develop research skills.

## Introduction

Chemistry laboratory experiments remain at the core of many undergraduate chemistry programs, but typical teaching practices can result in students learning disconnected pieces of knowledge lacking context to the relevance of chemistry to everyday life.^1,2^ The acquisition of knowledge through discrete facts makes it difficult for students to understand and interpret complex chemical systems and their properties. For example, analytical techniques are often taught as individual topics, and while this is important for gaining specific knowledge, students often lack training in how to correlate and analyze multiple data sets collated from different analytical techniques.^3,4^

Standard practical laboratories can represent an expository learning environment with predetermined and specified outcomes where students simply follow a procedure in a lab manual, favoring hands-on rather than minds-on learning.^5^ Laboratory practicals tend to follow traditional branches of inorganic, organic, physical, and analytical chemistry, which can lead to compartmentalizing knowledge and techniques to specific subdisciplines of chemistry.^6,7^ In contrast, nearly all aspects of chemistry research involve the incorporation of multiple techniques and methodologies. Confinement to traditional subdisciplines is a fading concept, especially through the lens of sustainable and green chemistry where a deeper, systems-thinking approach is required.^8−10^

The benefit of Undergraduate Research Experiences (UREs) as active- and inquiry-based pedagogies is well recognized in providing enhanced student learning experiences and increased student success through gaining technical skills, critical thinking, and personal skills such as communication.^11,12^ It can further impact student retention and career decisions and promote the uptake of students pursuing a Ph.D.^13−15^ While students at the latter stage of their degree program usually engage in a course-based undergraduate research experience (CURE),^16−18^ such as a research-based project as part of the curriculum, students earlier in their degree typically have a limited understanding and little to no experience of the research process.^19^ Internship-style immersive UREs where undergraduate students spend several weeks working within a research group, such as summer research placements, provide a formative experience at undergraduate level.^20^ Students typically benefit from one-to-one interaction with a Ph.D. or postdoctoral researcher and exposure to an interdisciplinary, collaborative learning experience that is very different from traditional undergraduate practical laboratory teaching. However, these schemes usually involve a small number of undergraduate students working with individual faculty members in their laboratories. Therefore, the development of initiatives such as summer cocurricular activities to expose a greater number of undergraduate students to the research process earlier in their undergraduate career has several benefits to student learning and builds student research skills.^21−24^

## Motivation and Objectives of Activity

The motivation for this activity was underpinned by an undergraduate student conducting an eight week URE in the form of a summer research internship. The question to be addressed was: How can an individual URE be redesigned to benefit a larger undergraduate audience? To address this, the objective was to design a research-focused activity in the form of a research-focused workshop involving a collaborative research experiment, where results were gathered collectively. The activity was carried out over the summer (3 weeks prior to the semester starting) and involved second- and third-year undergraduates of a four-year undergraduate program who had no prior experience in research-based laboratory activities. The workshop was an optional, nongraded cocurricular activity. The workshop was advertised by email to undergraduate students as an optional research experience workshop on sustainable chemistry and materials. The following description was provided to the students: “Participants will engage in experimental activities and analysis, discussions, and collaborative tasks related to sustainable materials. It offers a great opportunity to engage in scientific research and get an insight into what chemistry research is really like”. There were ten undergraduate participants, and the instructors of the activity were the undergraduate student undertaking the summer URE and a doctoral student. The activity was overseen by a faculty member. The activity duration was 2.5 h; however, 3 h was allowed overall for the workshop.

## Experimental Focus and Design

**Figure 1:**
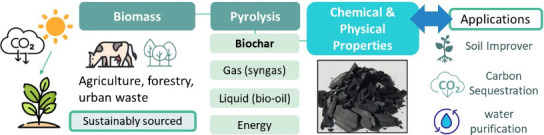
Schematic illustrating the preparation and applications of biochar from biomass. Shown to students as part of the introductory presentation.

Surface chemistry is a complex and nuanced field that often presents several misconceptions for students, such as assuming surfaces are ideally flat and homogeneous. Obtaining an accurate and comprehensive picture of the chemical functionality at surfaces and interfaces is challenging and often relies on analysis compiled from multiple techniques. Understanding the surface chemistry of bioderived materials further adds to this complexity due to the variability of biobased materials. However, the use of renewable feedstocks is a central concept to green and sustainable chemistry, and processing biomass feedstocks in a laboratory environment^25−28^ is an excellent opportunity to connect these principles, promote critical thinking, and engage students in systems thinking, which is typically not extensively taught in the undergraduate chemistry curriculum.^29−32^Figure 1 depicts the preparation process from biomass to biochar, emphasizing key applications and the significance of surface chemistry in these contexts.

In this context, biochar represents an excellent material as a platform to integrate systems thinking and inquiry-based learning to enhance students’ understanding of the interconnectedness and complexity of scientific and environmental problems and societal challenges in the development of sustainable materials.^33,34^ Biochar is a renewable feedstock, produced through the pyrolysis of biomass. The surface chemistry of biochar is rich in oxygen-containing functional groups such as carboxyl, hydroxyl, and phenolic groups. These groups play a key role in determining the performance and functionality of biochar, which can vary widely depending on the feedstock and pyrolysis conditions.^35^ While publications on biochar research are extensive, its use in undergraduate laboratory experiments is limited. Several laboratory experiments use biochar as an adsorbent and UV–vis spectroscopy to study adsorption of species such as methylene blue,^36,37^ salicylic acid and nitroaniline,^38^ and phosphates.^39^ This work differs from these experiments as the focus of this activity is on the surface characterization of biochar rather than on demonstrating a specific application of biochar. More significantly, this work describes a research experience workshop aiming to offer undergraduate students a more informative and realistic experience of research through working alongside a peer who had gained research experience during their URE. While other publications in this area typically emphasize student performance in laboratory reports, this initiative is aimed at inspiring undergraduates to pursue research opportunities and promote the development of research skills.

This activity uses the Boehm titration method as an analytical tool for the quantification of oxygen-containing surface groups that can be used for a wide variety of carbon materials. It is an attractive undergraduate experiment for practicing and reinforcing a variety of basic laboratory skills, e.g., potentiometric titrations, filtration, pipetting, pH paper, and reflux setup. The activity was designed as a collaborative experiment, where students used different biochars and the results were used collectively. In addition to this chemical method of surface characterization, several spectroscopy techniques are utilized in the activity for surface and chemical compositional analysis.

## Student Learning Objectives

- To acquire a more unified and integrated view of chemical knowledge to be able to build connections between central ideas in surface chemistry and sustainable materials.
- To gain an understanding of and engage in a collaborative experiment, as well as be involved in peer-to-peer teaching and group learning.
- Develop skills in interpreting spectroscopic data using various spectroscopic techniques and how to draw meaningful conclusions about material properties.
- Enhance problem-solving abilities by formulating hypotheses to address challenges encountered during research, such as unexpected results.
- Engage in critical thinking to address challenges and opportunities in the field of surface chemistry and sustainable materials.
- Gain experience in a research environment to consider pursuing postgraduate research after their undergraduate studies.

## Experimental Design and Activity

**Figure 2:**
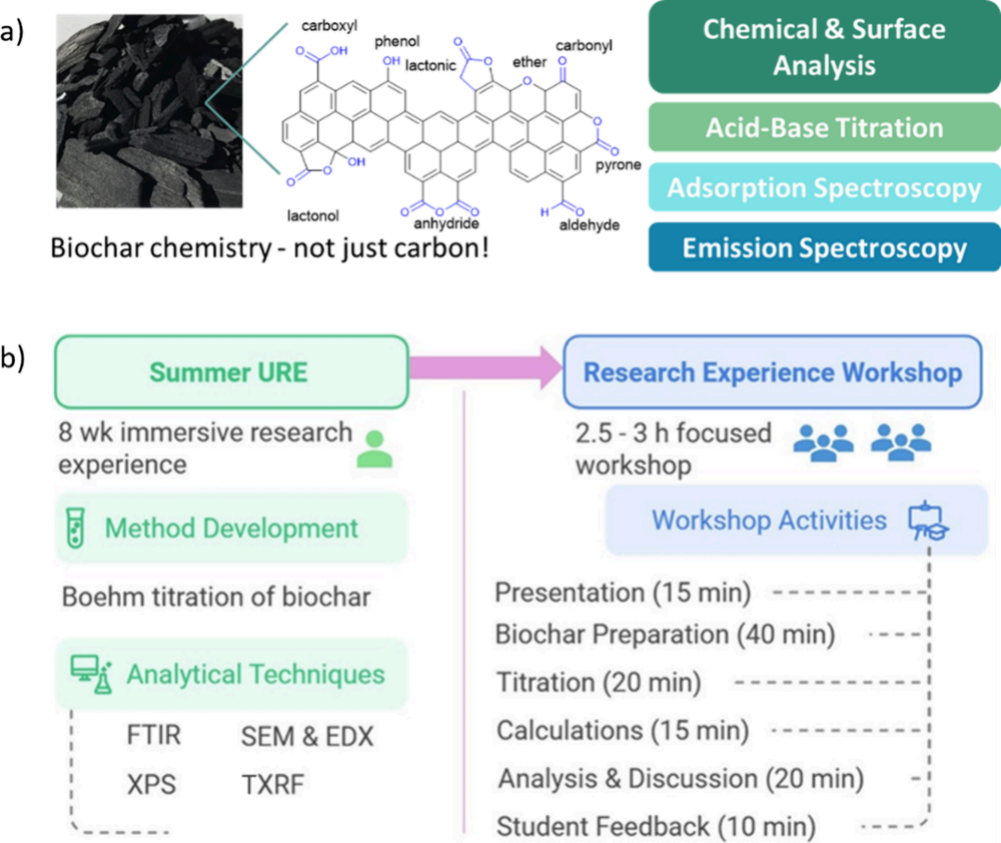
(a) Schematic highlighting the complex surface chemistry of biochar and activities that will be done in the workshop. (b) Schematic highlighting the connection between the activities of the URE and how they were redesigned as a shorter research experience workshop.

The activity reported here describes the research workshop that was carried out. Figure 2 illustrates the activities of the workshop and the connection between the URE and a breakdown of the workshop activities. We believe the concept of using UREs to design research-focused workshops could be adopted for other UREs such as course-based research projects.

See the Supporting Information for hazards associated with the experiment, detailed experimental descriptions, structure and flow schematic for the activity (Figure S1), instructor notes, practical considerations, and experimental modifications that can be used depending on time allocated and the skill level of the students. The activity was designed as a collaborative experiment, where students used different biochars, and the results were used collectively. Different student groups were assigned different biochar samples and bases, meaning that everyone’s data would collaboratively be used for the Boehm calculations.

The Boehm titration is a method used for the quantitative analysis of oxygen functional groups on a wide range of carbon materials.^40^ Specific oxygen-containing functional groups have varying acidities that can be selectively neutralized by different bases. However, using the Boehm titration for the analysis of biochars requires some caution, as biochar may also be composed of inorganic basic components such as oxides, hydroxides, and carbonates, as well as inorganic acidic species, e.g., humic-like structures, silica, and organic acids, that can be divergently solubilized in the Boehm bases and introduce inaccuracies into the titration, which is well discussed by Graber et al.^41^ To avoid this, biochars can be pretreated with acid and/or base washing prior to the titration. Four biochar samples were used in this activity: biochar derived from wood (WD) and biochar derived from digestate (DG), each prepared at temperatures of 300 °C (WD300 and DG300) and 700 °C (WD700 and DG700). Activated carbon was used as a reference carbon and as an example, where the results were already complete to show the students. The biochars were not pretreated prior to the Boehm titration, and the purpose of this was to intentionally return an unexpected result (a negative value) in the Boehm calculation, to promote problem solving in a research setting, and also to promote discussion of the results.

### Activity 1: Presentation

A short presentation was given to the students overviewing the aims of the workshop, describing what biochar is, and detailing the specific activities and analysis techniques that would be carried out in the workshop. One aspect highlighted in the presentation was that the biochar, although dominantly carbon, may contain other elements. We encouraged the students to think about the lifecycle of the material through the course of the activity.

### Activity 2: Preparation of Biochar for Assessment by the Boehm Method

**Figure 3:**
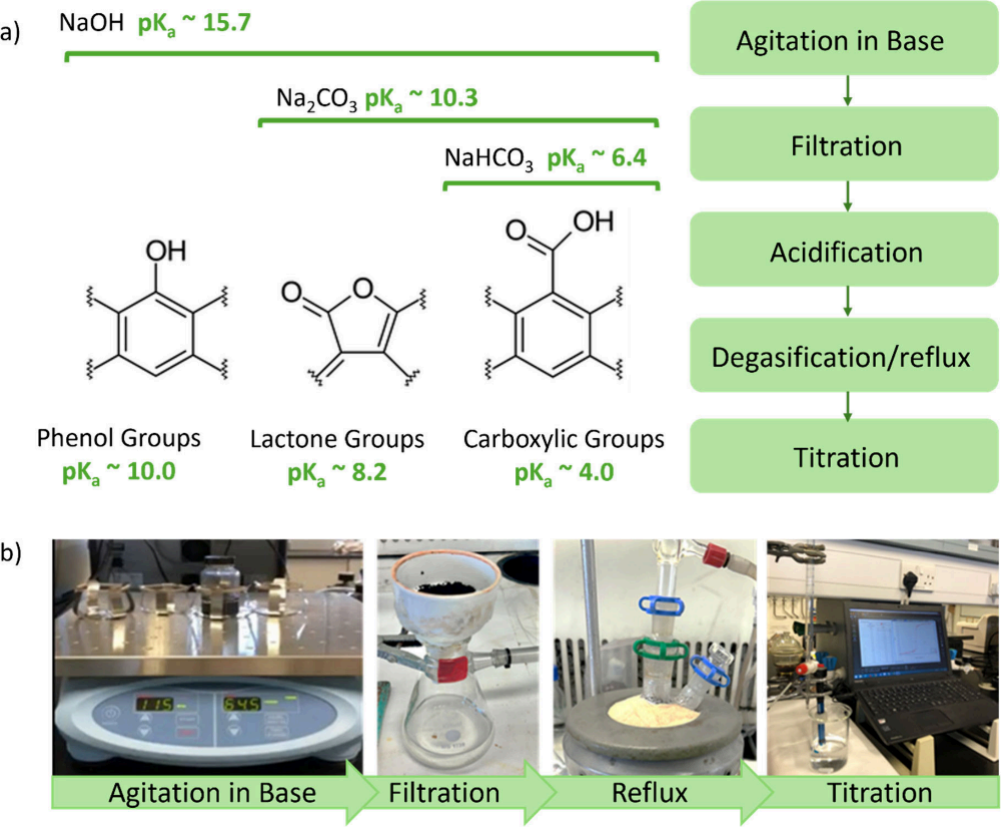
(a) Schematic illustrating the Boehm titration principle of selective neutralization of oxygen functional groups according to their acidity using multiple bases. (b) The flow process and pictures of the specific steps for the titration.

The Boehm titration works based on different oxygen-containing functionalities possessing different acidities and therefore can undergo selective neutralization with different bases, specifically sodium bicarbonate (NaHCO_3_), sodium carbonate (Na_2_CO_3_), and sodium hydroxide (NaOH). Carboxylic groups (p*K*_a_ = 3–6), lactonic groups (p*K*_a_ = 7–9), and phenolic groups (p*K*_a_ = 8–11) are the three main oxygen-containing surface functionalities of carbon materials, as illustrated in Figure 3.^31,34^

The first step of the Boehm titration is dispersion and agitation of the biochar in a base overnight (NaOH, NaHCO_3_, or Na_2_CO_3_); see the Supporting Information for a more detailed description. This is the only step of the Boehm titration that needs to be prepared beforehand. In the activity, the students still completed this step, i.e., they added the base to the dry biochar and placed in on the agitator (picture 1 in Figure 3) or stirrer but used a prepared biochar that had been left stirring overnight. The biochar was then filtered, and the liquid was collected (picture 2 in Figure 3).

The next step is acidification of the liquid fraction (see the Supporting Information for a detailed description). Briefly, 5 mL aliquots of the filtrate were transferred to 50 mL round-bottom flasks and acidified with 0.05 M HCl. The aim of this step is to add acid until a pH of 2 or 3 is achieved, indicating the HCl is in excess. This was achieved by adding different HCl volumes depending on the specific nature of the biochar. For the purposes of time, the students were told the volume of acid to add to the filtrate by providing them with a table. As shown in Table 1, 15 mL of 0.05 M HCl was initially added, followed by the addition of HCl in 5 mL increments, and the pH was tested with pH paper after each addition. Tabulating the data can help students to identify which biochar samples have more basic or acidic properties, initiating thought and discussion about the surface chemistry of the biochar during the experiment.

**Table 1:** Representative Student Results Showing pH Measurement Using pH Paper after Acidification of Samples Dispersed in NaOH, NaHCO_3_, and Na_2_CO_3_ Using 0.05 M HCl^a^

	base: NaOH				
biochar	AC	DG300	DG700	WD300^b^	WD700
total HCl (mL)	pH	pH	pH	pH	pH
15	3	8	7	3	5
20	2	3	4	3	4
25	2	3	3	3	3
30	2	2	2	3	3

	base: NaHCO_3_				
biochar	AC	DG300	DG700	WD300	WD700
total HCl (mL)	pH	pH	pH	pH	pH
15	3	7	8	4	5
20	3	4	6	3	4
25	3	3	4	3	3
30	3	2	3	3	3

	base: Na_2_CO_3_				
biochar	AC	DG300	DG700	WD300	WD700
total HCl (mL)	pH	pH	pH	pH	pH
15	8	8	8	8	8
20	7	8	7	7	8
25	5	8	7	6	7
30	3	7	6	4	6
35	3	5	5	3	5
40	3	3	4	3	4
45	3	3	3	3	3
50	3	3	3	3	3

a Sample Codes: AC = acidified activated carbon, DG300 = digestate biochar prepared at 300 °C, DG700 = digestate biochar prepared at 700 °C, WD300 = wood biochar prepared at 300 °C, WD700 = wood biochar prepared at 700 °C.

b Full sample calculation is given in the Supporting Information for WD300 biochar.

While the exact volume of acid added will depend on the biochar, one point worth highlighting is that biochar samples dispersed in monoprotic bases (NaOH and NaHCO_3_) will require less acid compared to samples dispersed in a diprotic base (Na_2_CO_3_). Based on the students’ knowledge of equilibrium constants for acids and base (typically covered in first year chemistry), students should be able to rationalize that as a diprotic base, in water, the carbonate ion (CO_3_^2–^) can undergo two sequential protonation steps.

Table 1 illustrates representative student data for acidification using different biochar samples. The final preparation step in the Boehm titration is degassing to remove dissolved CO_2_. This was carried out by doing a standard reflux setup, as shown in Figure 3. Bubbling inert gas through the solution can be done as an alternative to reflux for removing dissolved CO_2_.

### Activity 3: Boehm Titration

**Figure 4:**
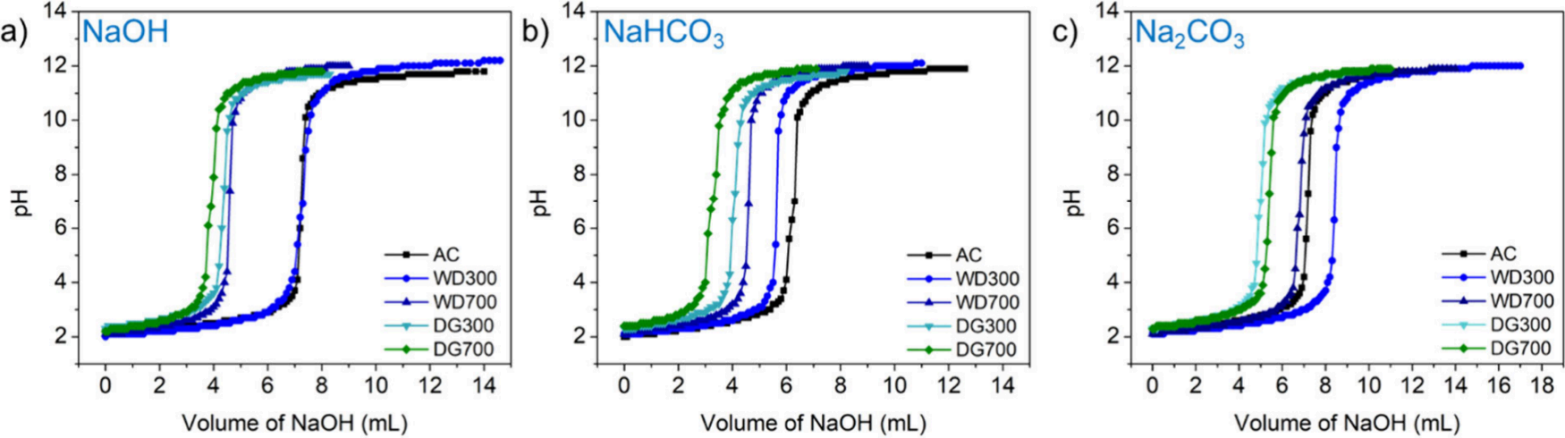
Titration curves for AC, WD300, WD700, DG300, and DG700 from agitation bases (a) NaOH, (b) NaHCO_3_, and (c) Na_2_CO_3_.

The acidified, degassed solutions were then titrated against standardized 0.05 M NaOH solution using a pH probe and Capstone PASCO software. Representative titration curves are shown in Figure 4, and the end point of each titriation is tabulated as shown in Table 2.

**Table 2:** Titration End Point Data Obtained from Different Biochar Samples

biochar sample	*V*_NaOH_ (mL)	*V*_NaHCO_3__ (mL)	*V*_Na_2_CO_3__ (mL)
AC	7.2	6.3	7.2
WD300^a^	7.3	5.6	8.4
WD700	4.6	4.6	6.8
DG300	4.4	4.0	4.9
DG700	3.9	3.3	5.4

a A sample calculation using these values is illustrated in the Supporting Information.

### Activity 4: Calculating Surface Functionalities

The students used the end point values determined from the titrations to calculate the amount of phenolic (Ar–OH), lactonic (−COOR), and carboxylic (−COOH) groups present on the biochar surface according to the equations adapted from Wu et al.^40^ The calculation can be divided into three parts, which include calculating (i) the volume of HCl that reacted in the acidification step (Δ*V*_HCl_; data from Table 1), (ii) the number of moles of each base that reacted during the agitation step (*n*) and (iii) concentration of oxygen functional groups (*C*). The equations are illustrated below, and the Supporting Information illustrates a completed sample calculation using data from Boehm titrations carried out with the WD300 biochar (data illustrated with a footnote in Table 1 and Table 2).

#### Volume of Reacted HCl (Δ*V*_HCl_)


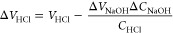
1where *V*_HCl_ is the volume of 0.05 M HCl used in the acidification step that is recorded in Table 2. Δ*V*_NaOH_ is the end point for the titration. *C*_NaOH_ is the concentration of NaOH in the titration (0.05 M). *C*_HCl_ is the concentration of HCl in the acidification step (0.05 M).

#### Number of Moles of Each Base (*n*)


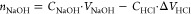
2a
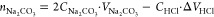
2b
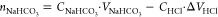
2cwhere *n* is the number of moles of base that reacted during the agitation step. *C*_NaOH_, *C*_Na_2_CO_3__, and *C*_NaHCO_3__ are the concentrations of the base solution used in the agitation step (0.05 M). *V*_NaOH_, *V*_Na_2_CO_3__, and *V*_Na_2_CO_3__ are the volumes of base used in the agitation step (0.025 L). *C*_HCl_ is the concentration of HCl used in the acidification step (0.05 M).

#### Concentration of Functional Groups (*C*)


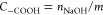
3a
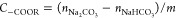
3b
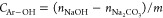
3cwhere *C* is the concentration of functional group, *m* is the mass of carbon used in the dispersion and agitation steps, and *n* is the number of moles of base that reacted during the agitation step.

### Activity 5: Spectroscopic Analysis Using FTIR

**Figure 5:**
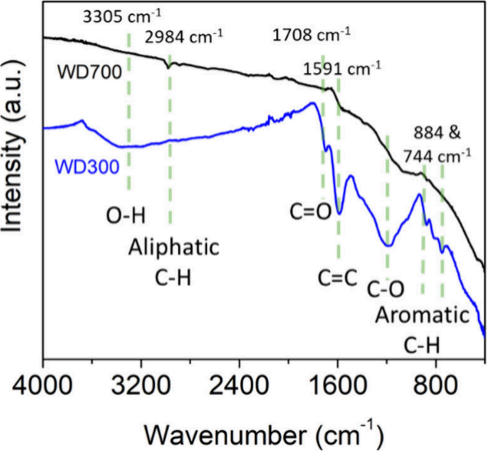
FTIR analysis of wood-derived biochar prepared at 300 and 700 °C.

Figure 5 illustrates representative FTIR spectra of the wood-derived biochars collected by the students during the workshop. All of the students had previous experience in collecting FTIR data as well as theoretical knowledge of FTIR. The presence of the oxygen-containing functional groups can be readily observed in the WD300 biochar and not very well resolved for the other biochars. The specific surface chemistry is highly dependent on the feedstock and preparation conditions, but in general biochar prepared at higher annealing temperatures has a higher degree of aromatization and a lower amount of oxygen containing functionalities, which can be clearly observed in comparing the WD300 biochar to the WD700 sample in Figure 5.^42^ The FTIR analysis of the digestate samples is shown in the Supporting Information, Figure S2.

### Activity 6: Group Discussion

The group discussion was carried out in parallel with an additional chemical analysis of the biochar. Further details of how this was managed are outlined in the Supporting Information. The Boehm titration quantifies carboxylic, lactonic, and phenolic groups in carbon samples, and sample results obtained from collaborative analysis of different biochars are shown in Table 3. It was found that all biochar samples contained a large amount of lactonic groups, with the WD300 biochar containing the highest concentration. The WD300 sample was the most similar to the acidified activated carbon sample as it also contained carboxylic groups. This result correlated well with the FTIR analysis and reflects the oxygen rich chemistry expected for a wood-sourced biochar sample and low annealing temperature. The effect of the preparation temperature is illustrated in the wood-derived biochar as the WD700 biochar showed a significant reduction in the carboxylic acid groups, which further correlates the Boehm results with the FTIR analysis.

**Table 3:** Concentration of Different Oxygen Containing Functional Groups Determined Using the Boehm Method for Each Sample^a^

biochar sample	*C*_–COOH_ (μmol g^–1^)	*C*_–COOR_ (μmol g^–1^)	*C*_Ar–OH_ (μmol g^–1^)
AC	87	387	0
WD300^b^	430	530	–420
WD700	–27	480	–480
DG300	–53	386	–286
DG700	–70	476	–430

a See the Supporting Information for the worked sample calculation.

b The worked sample calculation using these values is illustrated in the Supporting Information.

An apparently anomalous result obtained from the Boehm method is the negative values obtained from the phenolic groups and to a lesser extent the carboxylic acid groups, except for WD300, which showed a positive value. These negative values are not observed with the commercial activated carbon. Students were asked if they had any theories on what this could mean, thinking about the calculations used in the experiment and the acid–base nature of the titration used for the Boehm method.

As part of the discussion, students were reminded of the schematic in Figure 3 illustrating the Boehm titration principle of selective neutralization of oxygen functional groups according to their acidity: (i) the NaOH will accept protons from all Brønsted acid species (carboxylic acids and phenols), (ii) the Na_2_CO_3_ will accept protons from functionalities with p*K*_a_ values <10.3, and (iii) NaHCO_3_ will accept protons from functionalities with p*K*_a_ values <6.4. The application of the Boehm method to biochar can introduce biases to the method through the following mechanisms: (i) dissolution of the inorganic or ash fraction with the Boehm reactants would contradict the premise that the reactants will only have interations with organic surface functional groups, and (ii) reactive biochar components containing acidic or basic groups may dissolve when reacted with the Boehm reagents, especially during the base dispersion step, forming dissolved organic carbon (DOC).^43^ The generation of negative values using the Boehm titration is well reported in biochar due to various mechanisms, such as the inorganic components in the biochar leading to unexpected results.^40^

In the initial presentation, it was suggested that noncarbon elements may be present in biochar samples from different sources. Students were not told specifically what noncarbon species the biochars contained but rather that they should consider the biomass used of the preparation of the biochar material and the life cycle of the material. Students were asked to reflect on this and to think about what species might be present in the biochar sample. Most students were able to make the connection between the presence of chemical species that could give rise to the unexpected titration results. The elements most identified by students were potassium, phosphorus, and nitrogen, as these are present in fertilizer. To further rationalize the results obtained from the Boehm method, chemical compositional analysis of the biochar was carried out to support the Boehm results.

### Activity 7: Chemical Analysis of Biochar

**Figure 6:**
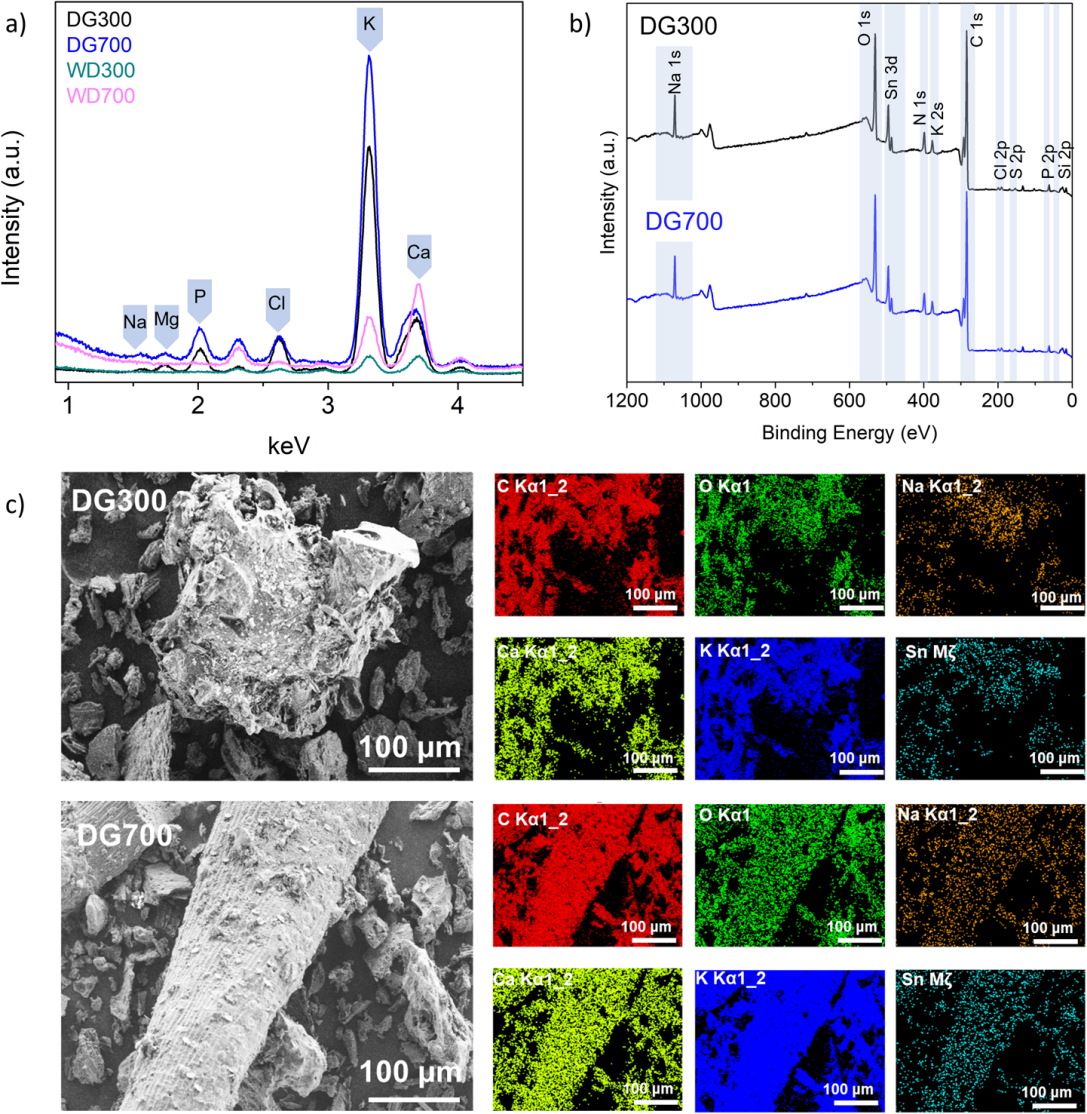
(a) TXRF scan of biochars samples DG300, DG700, WD300, and WD700. (b) XPS survey of DG300 and DG700 samples. (c) SEM with EDX mapping of the DG300 and DG700 samples.

Chemical compositional analysis of the biochar was carried out using total reflection X-ray fluorescence (TXRF). TXRF is a fast (typical scan <60 s) and easy nondestructive method for the students to measure the elemental composition of a material. Figure 6a displays representative spectra of the digestate biochars and confirms the presence of trace minerals within the sample, and the absolute values generated from the S2 Picofox software (μg) are shown in Table 4 (see the Supporting Information for details). DG300 had the highest and most diverse mineral content and showed the presence Na, Mg, P, Cl, K, and Ca, which is to be expected as digestate is nutrient-rich. It is interesting to note the P to K ratio is about 1:3, indicating that the digestate biochar has potential as an agricultural soil improver. The DG700 biochar only showed the presence of K, suggesting other minerals were volatized at the higher preparation temperature. In contrast to the digestate derived biochar, the wood-derived biochar showed a much lower total mineral content and was more similar at different synthesis temperatures, as shown in Table 4. Shown in Figure S3, the AC reference material showed no signals in the TXRF scan, as expected.

**Table 4:** TXRF Data Measured as Absolute Values Generated from the S2 Picofox Software

sample	Na (μg)	Mg (μg)	P (μg)	Cl (μg)	K (μg)	Ca (μg)	total (μg)
DG300	2.06	0.07	0.12	0.07	0.18	0.03	2.53
DG700					0.28		0.28
WD300			0.002		0.01	0.01	0.022
WD700				0.003	0.02	0.03	0.053

While TXRF is a fast and convenient analytical method to identify chemical composition, it is unsuitable for light elements, and spectroscopic techniques such as XPS and EDX are commonly employed in surface and chemical analysis.^44^ This analysis was gathered by the undergraduate student doing the Summer URE, and while the students of the workshop could not obtain hands-on experience in gathering the XPS and EDX analysis, it nonetheless is an excellent opportunity to demonstrate (i) the use of correlative analysis in research to draw more meaningful conclusions,^45,46^ (ii) the fundamental operating principles of different spectroscopic techniques, and (iii) that specific methodologies have advantages and disadvantages that need to be considered when selecting an analysis method.

Figure 6b shows the XPS survey spectra of the digestate biochar that were shown to the students. In addition to the surface sensitivity of XPS, it is also suitable for the analysis of C, O, and N. It can be readily seen that the XPS analysis correlated with the TXRF analysis, also showing inorganic species, Na, K, Cl, and P. EDX mapping (Figure 6c) provides spatial information about the location of the specific elements and again correlates well with TXRF and XPS analysis.

The chemical analysis data provided a good basis for a group discussion. The detection of nitrogen groups in the biochar is significant as they will contribute to the basicity of the surface (ammonia, ammonium ions, amine, and amide functional groups) of the biochar, resulting in the overconsumption of HCl in the acidification step prior to titration, which further supports the results obtained through the Boehm titration. Students were asked to theorize the origin of these elements, thinking about the material originating from biomass and anaerobic digestion. One interesting element detected in the digestate biochar was tin. While the origin of the Sn is unclear, it served as a useful result for students to think more holistically beyond the analysis and consider the life cycle of the material from its starting point as biomass to production into biochar, taking into account it may be a contaminant in the biomass or was introduced at storage, the preparation stage, or even a cross-contamination in the lab.

Overall, the additional characterization of the biochar reveals the complex chemistry of the biochar structure and supports an explanation for the Boehm titration results. This led to a discussion on the need to critically assess the Boehm method, a methodology originally designed for pure carbons, when applying it to biochar. Finally, we discussed how the biochar could be pretreated to remove the noncarbon components. Pretreating the biochar with NaOH can remove any solubilizable acidic functionalities, and then treatment with HCl can remove solubilizable basic functionalities.

## Student Feedback

**Figure 7:**
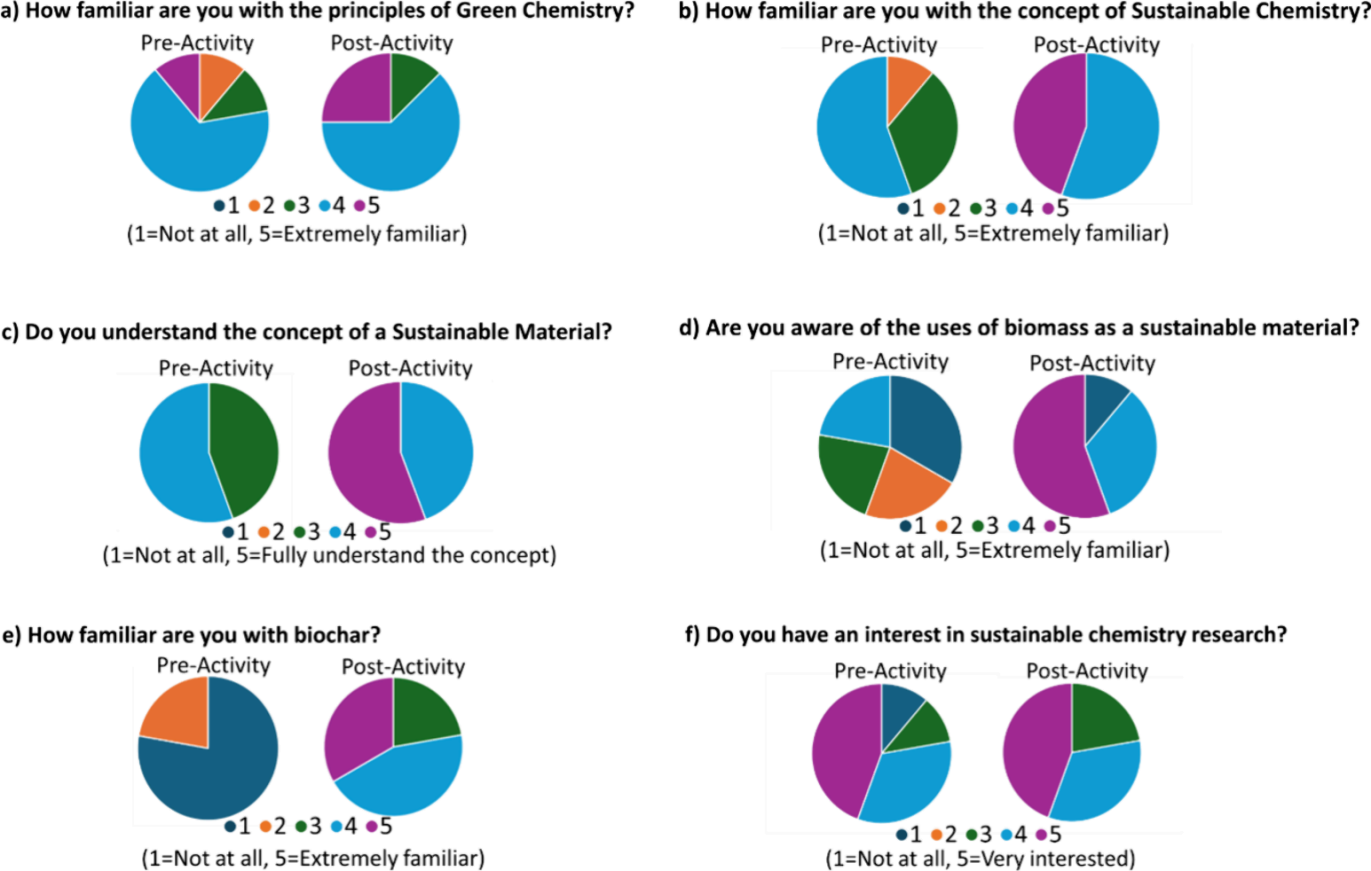
Summary of the pre- and post-activity survey (10 respondents).

A student survey was carried out pre- and postactivity, and the results are displayed in Figure 7. Students were not familiar with biochar as a material and had a poor understanding on the concept of sustainable materials. Student feedback showed improvement in understanding, and comments from student survey evidenced that students enjoyed the interaction with the doctoral students and obtained a better understanding of the research process. Specific student comments demonstrated increased motivation in pursuing further research.

The survey also had an open comment box for student input. Below are quotes taken from the student survey. The positive feedback comments received from the students indicate a better understanding and appreciation of the research process. While the survey was largely qualitative, it nonetheless provides supporting evidence that the key objectives of this activity were achieved.

*“Going in I was sure I wanted an industry placement but now I’m also considering research thanks to the workshop, which piqued my interest in research.”*

*“Enjoyable lab, learned a lot and gave insight into what research is like.”*

*“Great workshop. Real life application of undergraduate experiments.”*

*“Really enjoyed seeing the research side of chemistry.”*

## Pedagogical Reflection

The use of biochar as a renewable feedstock provides a genuine inquiry-based laboratory activity that provides students the opportunity to engage in an investigative research process. It highlights that investigative methodologies are not a “one size fits all” and that methodologies can have bias depending on the nature of the substance being analyzed. For example, the Boehm titration returned a typical result for activated carbon, but the biochar samples gave rise to unexpected results that required further investigation. Rationalizing these results by using different chemical analysis techniques enabled students to build a wider picture of the chemical complexity of biochar and how the use of multiple analytical techniques contributed to a better understanding of material properties and promoted research skills. Students also benefited from the collaborative nature of the workshop, where they collectively contributed to the experimental results and discussion. While the activity was designed as a research experience, the activity could be easily adapted to a conventional laboratory experiment. The Supporting Information outlines practical considerations, and Modifications to the Experiment offers several suggestions to modify the experiment.

## Conclusions

Cocurricular research experiences provide valuable opportunities for students to develop their scientific research skills and gain insight into the research process. This activity developed a succinct research-based workshop from a longer URE, in this case an undergraduate doing a summer internship. This approach has many advantages, including enabling a greater number of students to experience research and promoting peer-led teaching and group learning. This activity was conducted outside of the semester, but we believe this approach could also be applied to curriculum based UREs where research-orientated workshops could be designed from individual UREs with a focus on developing UG research skills.

## Supporting Information Available

Notes for instructor, experimental description sample calculation, blank data table templates, FTIR analysis, TXRF analysis, practical considerations and modification to the experiment, extension to the experiment, and supplementary data (PDF)
Notes for instructor, experimental description sample calculation, blank data table templates, FTIR analysis, TXRF analysis, practical considerations and modification to the experiment, extension to the experiment, and supplementary data (DOCX)
