# Supplementary material for: A Collaborative Cocurricular Undergraduate Research Experience on Sustainable Materials: Analysis of Biochar Using the Boehm Titration and Spectroscopic Techniques
Source: J Chem Educ. 2025 Feb 7;102(3):1323–32. doi: 10.1021/acs.jchemed.4c01110 (PMC11905283; doi:10.1021/acs.jchemed.4c01110)
Supplement: Supplementary file 1 — ed4c01110_si_001.pdf [file ed4c01110_si_001.pdf]

A Collaborative Co-Curricular Undergraduate Research Experience on Sustainable Materials: Analysis of Biochar using the Boehm Titration and Spectroscopic Techniques

Supporting Information

Rachel Breen[1,2], Conor Goggin[1], Justin Holmes[1,2] and Gillian Collins*[1,2]

[1] School of Chemistry, University College Cork, Cork, T12 YN60, Ireland.

[2] AMBER Centre, Environmental Research Institute, University College Cork, Cork, T23 XE10, Ireland.

Notes for Instructor

1. Experimental Description:
2. Sample Calculation
3. Blank Data Table Templates
4. FTIR Analysis
5. TXRF Analysis
6. Practical Considerations and Modification
7. Extension to the Experiment

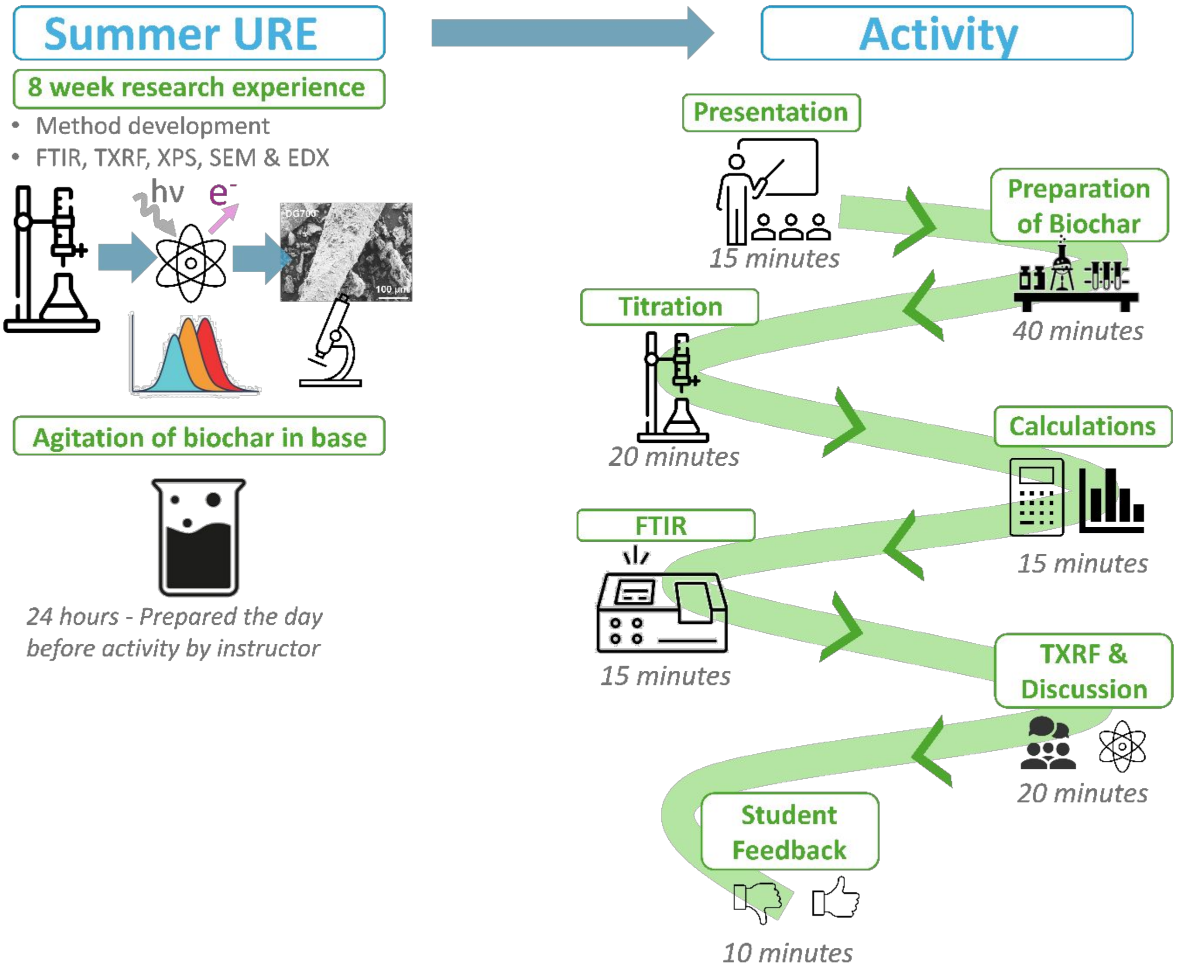

Figure S1: Class structure and flow schematic for the activity.

Experience

1. Experimental description

Hazards

This experiment presents low degree of the hazard including mild irritants and corrosives (dilute NaOH & HCl solutions). Standard safety precautions and proper PPE, lab coat, glasses and gloves should be used when performing this activity.

Boehm Preparation and Titration

1.1 Dispersion and Agitation of Biochar in Base.

This process is conducted prior to students' arrival. 0.75 g of biochar was added to a 100 mL jar containing 25 mL 0.05 M of NaOH, $NaHCO_3$ or Na_2CO_3. Sample dispersion was achieved through 24 h

of shaking at 115 rpm. In the workshop students still completed this step of the process *i.e.* they pipetted the base and added it to dry biochar and put it on the agitator but used one of the overnight samples for their experiments.

Shaking was used as the form of agitation as high-speed magnetic stirring has been shown to disrupt the structure of the carbon surface, however a standard stirrer on a low rpm setting could also be used.[1] The screw-top jar lids were left on to avoid contamination, but loose to account for any possible gas production leading to pressure build-up. This long-period of agitation allowed for the bases to react with any surface acidic functional groups. The resulting mixture consisted of H_2O from neutralized functional groups, dissolved CO_2, and an unknown amount of unreacted base. This mixture was filtered by Buchner filtration to remove any solid carbon from the liquid fraction.

1.2 Acidification of Liquid Fraction

In this step the excess HCl reacted with any residual base in solution forming an acidic mixture. 5 mL aliquots of the filtrates from part 1 were transferred to 50 mL round bottom flasks and acidified with 0.05 M HCl to achieve a pH of 2-3 which is tested using pH paper. The amount of acid that will be required will depend on the specific biochar. It is not necessary to provide students with the volume of acid to, but for this activity the students were provided with a table specifying the volume, shown in the Table 1 in the main paper. As shown in Table 1, main paper, 15 mL of HCl was first added, followed by 5 mL aliquots of HCl. The pH of the solution was tested after each addition with pH paper and recorded in the Table 1. [2] Acidified activated carbon was used as a reference material. The resulting pH of all mixtures was between 2 and 3 which was indicative that HCl was present in excess.

1.3 Degasification of Acidified Sample

The round bottom flasks were placed in a heating mantle and refluxed for 30 minutes at 100 °C with stirring at 300 rpm.[3] This step allows for the removal of any dissolved CO_2 from the samples which would have further acidified the samples affecting titration results. After the reflux period the flasks were sealed and left to cool to room temperature.

1.4 Titration of Degassed Sample against NaOH

Once cool, the samples were titrated against 0.05 M NaOH solution using a pH probe and capstone PASCO software. For complete accuracy, the NaOH solution can be standardised by titration with potassium hydrogen phthalate prior to titration. Sample titration curves obtained are shown in the main text. Our activity used potentiometric titrations but a standard pH procedure with an indicator such as phenolphthalein can be used as an alternative.

2. Blank Data Tables

The students were provided with the tables below to complete during the activity. AC was used as a reference and to show the students an example of the data that was complete.

Acidification tables:

	**Base: NaOH**				
**Biochar**	**AC**	**DG300**	**DG700**	**WD300**	**WD700**
**Total HCl (mL)**	**pH**	**pH**	**pH**	**pH**	**pH**
15	3				
20	2				
25	2				
30	2				
	**Base: $NaHCO_3$**				
**Biochar**	**AC**	**DG300**	**DG700**	**WD300**	**WD700**
**Total HCl (mL)**	**pH**	**pH**	**pH**	**pH**	**pH**
15	**3**				
20	3				
25	3				
30	3				
	**Base: Na_2CO_3**				
**Biochar**	**AC**	**DG300**	**DG700**	**WD300**	**WD700**
**Total HCl (mL)**	**pH**	**pH**	**pH**	**pH**	**pH**
15	8				
20	7				
25	5				
30	3				
35	3				
40	3				

45	3				
50	3				

Titration table:

**Biochar Sample code**	$\boldsymbol{V_{NaOH}}$ ***(mL)***	$\boldsymbol{V_{NaHCO_3}}$ ***(mL)***	$\boldsymbol{V_{Na_2CO_3}}$ ***(mL)***

3. Worked Sample Calculation for Oxygen Surface Functionalities

The students used the end point values from the titrations to calculate the content of phenolic (Ar-OH), lactonic (-COOR) and carboxylic (-COOH) groups present on the biochar surface. These were calculated according to the equations adapted from Wu *et al.*[4]

A sample calculation using the Boehm titrations conducted with results from WD300 sample is outlined below:

Results for WD300 for acidification step:

**Base** (25 mL of 0.05 M base added to biochar)	**Volume of 0.05 HCl (V_{HCl}) added to reach pH 2/3 (mL)**
NaOH	30
$NaHCO_3$	30
Na_2CO_3	50

Results for WD300 for titration step:

Biochar Sample	$\boldsymbol{V_{NaOH}}$ (mL)	$\boldsymbol{V_{NaHCO_3}}$ (mL)	$\boldsymbol{V_{Na_2CO_3}}$ (mL)
WD300	7.3	5.6	8.4

Base: NaOH

(i) Volume of Reacted HCl, ΔV_{HCl}:

$$\Delta V_{HCl} = V_{HCl} - \frac{\Delta V_{NaOH} \cdot C_{NaOH}}{C_{HCl}}$$

$$\Delta V_{HCl} = 0.03\,L - \frac{0.0073\,L \cdot 0.05\,\mathrm{M}}{0.05\,\mathrm{M}}$$

$$\Delta V_{HCl} = 0.0227\,\mathrm{L}$$

(ii) Moles NaOH, n_{NaOH}:

$n_{NaOH} = C_{NaOH} \cdot V_{NaOH} - C_{HCl} \cdot \Delta V_{HCl}$

$n_{NaOH} = 0.05\,M \cdot 0.025\,\mathrm{L} - 0.05\,M \cdot 0.0227\,\mathrm{L}$

$n_{NaOH} = 1.15 \times 10^{-4}\,\mathrm{mol}$

Base $NaHCO_3$:

(i) Volume of Reacted HCl, ΔV_{HCl}:

$$\Delta V_{HCl} = V_{HCl} - \frac{\Delta V_{NaOH} \cdot C_{NaOH}}{C_{HCl}}$$

$$\Delta V_{HCl} = 0.03\,L - \frac{0.0056\,\mathrm{L} \cdot 0.05\,\mathrm{M}}{0.05\,\mathrm{M}}$$

$$\Delta V_{HCl} = 0.0243\,\mathrm{L}$$

(ii) Moles $NaHCO_3$, n_{NaHCO_3}:

$n_{NaHCO_3} = C_{NaHCO_3} \cdot V_{NaHCO_3} - C_{HCl} \cdot \Delta V_{HCl}$

$n_{NaHCO_3} = 0.05\,\mathrm{M} \cdot 0.025\,\mathrm{L} - 0.05\,M \cdot 0.0243\,\mathrm{L}$

$n_{NaHCO_3} = 3.25 \times 10^{-5}\,\mathrm{mol}$

Base Na_2CO_3:

(i) Volume of Reacted HCl, ΔV_{HCl}:

$$\Delta V_{HCl} = V_{HCl} - \frac{\Delta V_{NaOH} \cdot C_{NaOH}}{C_{HCl}}$$

$$\Delta V_{HCl} = 0.05\text{ L} - \frac{0.0085\text{ L} \cdot 0.05\text{ M}}{0.05\text{ M}}$$

$$\Delta V_{HCl} = 0.0415\text{ L}$$

(ii) Moles Na_2CO_3, $\boldsymbol{n_{Na_2CO_3}}$:

$n_{Na_2CO_3} = 2C_{Na_2CO_3} \cdot V_{Na_2CO_3} - C_{HCl} \cdot \Delta V_{HCl}$

$n_{Na_2CO_3} = 2 \cdot 0.05\text{ M} \cdot 0.025\text{ L} - 0.05\ M \cdot 0.0415\text{ L}$

$n_{Na_2CO_3} = 4.3 \times 10^{-4}\text{ mol}$

Concentration of Functional Group, $\boldsymbol{C}$:

Carboxylic groups, -COOH:

$C_{-COOH} = n_{NaOH}/m$

$C_{-COOH} = 1.15 \times 10^{-4}\text{ mol / }0.75\text{ g}$

$\boldsymbol{C_{-COOH} = 4.3 \times 10^{-5}\ \textbf{mol g}^{-1}}$

Lactonic groups, -COOR:

$C_{-COOR} = (n_{Na_2CO_3} - n_{NaHCO_3})/m$

$C_{-COOR} = (4.3 \times 10^{-4}\text{ mol} - 3.25 \times 10^{-5}\text{ mol})/\ 0.75\text{ g}$

$\boldsymbol{C_{-COOR} = 5.3 \times 10^{-4}\ \textbf{mol g}^{-1}}$

Phenolic, Ar-OH

$C_{Ar-OH} = (n_{NaOH} - n_{Na_2CO_3})/m$

$C_{Ar-OH} = (1.15 \times 10^{-4}\text{ mol} - 4.3 \times 10^{-4}\text{ mol})/0.75\text{ g}$

$\boldsymbol{C_{Ar-OH} = -4.2 \times 10^{-4}\ \textbf{mol g}^{-1}}$

4. FTIR Analysis

FTIR analysis was carried out using a Perkin Elmer Spectrum Two FT-IR Spectrometer in the range of 4000-450 cm^{-1} with a resolution of 4 and an average of 20 scans.

5. TXRF Analysis

Total reflection X-ray fluorescence spectroscopy was (TXRF) was carried out using an S2 Picofox TXRF Spectrometer with a scan time of 250 s. Absolute quantification was used to approximately quantify the inorganic components in the biochar. This quantification type is based on the measured absolute sensitivity for Ni for the instrument and is approximated by the software according to the following equation:

$$m_i = \frac{N_i \cdot S_{Ni}}{S_i \cdot s_{Ni} \cdot T \cdot I}$$

Where m_i is the absolute amount of an analyzed element within the sample, N_i is the net pulse number within the measurement spectrum of the analyte, S_{Ni} is the relative sensitivity of Ni, S_i is the relative sensitivity of the analyte, s_{Ni} is the absolute sensitivity of Ni in counts ($s^{-1}ng^{-1}mA^{-1}$), T is the measurement time and I is the tube current in mA. (adapted from the Bruker S2 Picofox TXRF manual)

6. Practical Considerations and Modification to the Experiment

- A key objective in this activity was to demonstrate that the nature of the biomass feedstock has an impact on the biochar that is produced and therefore four biochar samples were used. Feedstock specific biomass can be purchased from chemical suppliers. Biochar can also be purchased in gardening and horticulture stores. If it is not possible to obtain different types of biochar the activity could be run with a single sample and pretreated with acids or bases to change the surface chemistry.
- Commercial acidified activated carbon was used a reference, and results were shown as an example. This was a helpful guide to students, however activated carbon could be used as a specimen sample and then compared with biochar sample. Another alternative if it is not possible to obtain biochar is comparing commercial activated carbon and acidified activated carbon by stirring in nitric acid overnight.

- The degassing of the biochar sample takes 30 minutes. During this period FTIR analysis and standardization of the NaOH to be used in the titration could be carried out however this period could also be used for discussion.
- It is not possible to distinguish between the biochar samples so sample labelling at each step e.g. the Buchner funnels after filtration and beaker used for the acidification step is very important.
- The most effective method of degasification reported is direct bubbling nitrogen through the solution[3], however, for the purpose of undergraduate activity and accessibility, reflux is sufficient in this case.
- Prior to the Boehm titration, biochar can be pretreated to remove the inorganic components if desired. Pretreating the biochar with NaOH can remove solubilizable acidic species, and then with HCl to remove solubilizable basic components. This was not carried out in the activity as the intention was to illustrate the impact of the inorganic species on the Boehm method and engage student thinking and promote discussion on chemical analysis.
- Where a pH probe and appropriate software are not available, a standard titration method using a chemical indicator such as phenolphthalein could be used to determine the end point. A agitator was used to stir the biochar overnight but a stirrer plate could also be used.
- TXRF was used in this experiment as it is a user friendly, benchtop instrument with fast scan speeds. It is a commonly used instrument in wastewater and soil analysis and there may be scope for borrowing the instrument from other research groups. Other chemical analysis methods of biochar sample could be used inductively coupled plasma-mass spectrometry (ICP-MS).

  The final discussion and TXRF analysis were carried out in parallel. In the initial presentation, the students were shown slides about biochar from different sources, highlighting non-carbon elements that may be present in such samples. During the discussion, the students were asked to reflect on this and propose some elements they may think come from wood and digestate-derived biochars. In this time the concept of TXRF analysis was introduced to screen for inorganic components in the samples. While the TXRF scan was running, the URE and doctoral student presented XPS and EDX findings they had collected over the summer, showing the UG students the elemental composition of the digestate biochars, and demonstrating a variety of techniques that can be used to characterize a biomass-derived material.

A schematic of the class structure and flow with time approximations is shown below in **Figure S1**.

Extension to the Experiment

One extension to this activity that is particularly valuable would be to include error analysis. Error analysis is not always considered in undergraduate practical laboratory. In a research setting, these titrations should be carried out in at least duplicates or triplicates for accuracy and to allow for error. This was highlighted to the students during the workshop but not done, however if the activity was reduced to one biochar sample, this could be done. An error analysis was carried out by the undergraduate undertaking the URE and is described below. The average values and standard deviations for the titrations can be calculated as follows:

Average value, $\bar{n}$:

$$\bar{n} = \frac{n_1 + n_2}{2}$$

Where n_1 and n_2 are the moles of base calculated from the end point from titrations 1 and 2, respectively.

Standard Deviation, σ_n:

$$\sigma_n = \sqrt{\frac{(n_1 - \bar{n})^2 - (n_2 - \bar{n})^2}{2 - 1}}$$

Concentration of Functional Groups, *C*:

$$C_{-COOH} = \bar{n}_{NaHCO_3} / m$$

$$C_{-COOR} = (\bar{n}_{Na_2CO_3} - \bar{n}_{NaHCO_3}) / m$$

$$C_{Ar-OH} = (\bar{n}_{NaOH} - \bar{n}_{Na_2CO_3}) / m$$

Where *m* is the mass of carbon used in the dispersion step.

Standard Deviation of Functional Groups, *σ*:

$$\sigma_{-COOH} = \frac{\sigma_{n_{NaHCO_3}}}{m}$$

$$\sigma_{-COOR} = \frac{\sqrt{\sigma^2_{n_{NaHCO_3}} + \sigma^2_{n_{Na_2CO_3}}}}{m}$$

$$\sigma_{Ar-OH} = \frac{\sqrt{\sigma^2_{n_{NaOH}} + \sigma^2_{n_{Na_2CO_3}}}}{m}$$

Supplementary Data:

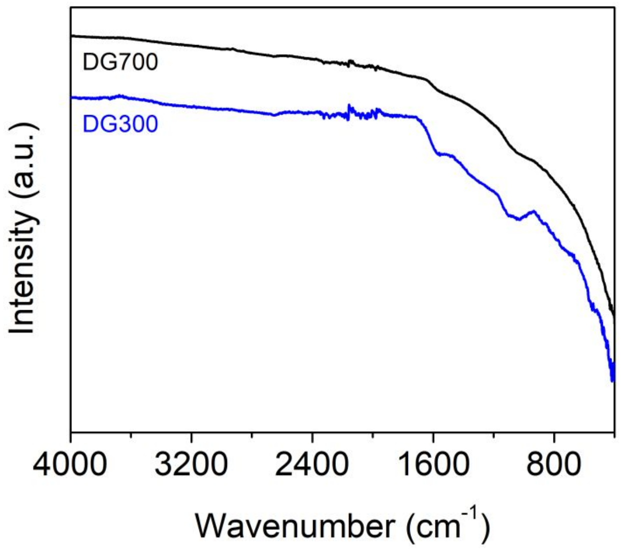

Figure S2: FTIR analysis of the digestate biochar prepared at 300 °C and 700 °C.

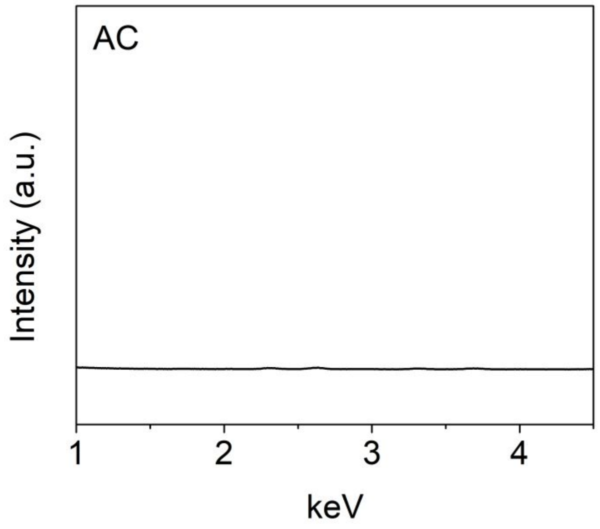

Figure S3: Reference TXRF of AC.

References:

(1) Oickle, A. M.; Goertzen, S. L.; Hopper, K. R.; Abdalla, Y. O.; Andreas, H. A. Standardization of the Boehm titration: Part II. Method of agitation, effect of filtering and dilute titrant. *Carbon* **2010**, *48* (12), 3313-3322. DOI: https://doi.org/10.1016/j.carbon.2010.05.004.

(2) Ren, H.; Cunha, E.; Sun, Q.; Li, Z.; Kinloch, I. A.; Young, R. J.; Fan, Z. Surface functionality analysis by Boehm titration of graphene nanoplatelets functionalized via a solvent-free

cycloaddition reaction. *Nanoscale Advances* **2019**, *1* (4), 1432-1441, 10.1039/C8NA00280K. DOI: 10.1039/C8NA00280K.

(3) Goertzen, S. L.; Thériault, K. D.; Oickle, A. M.; Tarasuk, A. C.; Andreas, H. A. Standardization of the Boehm titration. Part I. CO2 expulsion and endpoint determination. *Carbon* **2010**, *48* (4), 1252-1261. DOI: https://doi.org/10.1016/j.carbon.2009.11.050.

(4) Wu, H.; Lu, W.; Chen, Y.; Zhang, P.; Cheng, X. Application of Boehm Titration for the Quantitative Measurement of Soot Oxygen Functional Groups. *Energy & Fuels* **2020**, *34* (6), 7363-7372. DOI: 10.1021/acs.energyfuels.0c00904.
